# Authentic Consumer–Clinician Co‐Design of a Labour and Childbirth Intervention to Optimise Maternal Hydration in Labour: Reaching Consensus to Enhance Rigour

**DOI:** 10.1111/hex.70438

**Published:** 2025-10-03

**Authors:** Lauren Kearney, Bec Jenkinson, Christoph Lehner, Victoria Eley, Nicole Marsh, Deanne August, Susan de Jersey, Nigel Lee

**Affiliations:** ^1^ The University of Queensland Brisbane Queensland Australia; ^2^ Women's and Newborn Services, Royal Brisbane and Women's Hospital, Metro North Health Brisbane Queensland Australia; ^3^ Department of Anaesthesia and Perioperative Medicine Royal Brisbane and Women's Hospital, Metro North Health Brisbane Australia; ^4^ Nursing and Midwifery Research Centre, Royal Brisbane and Women's Hospital, Metro North Health Brisbane Australia; ^5^ Dietetics and Foodservices Royal Brisbane and Women's Hospital, Metro North Health Brisbane Australia

## Abstract

**Introduction:**

Medical interventions during labour and childbirth have rarely been designed with end‐users, including frontline clinicians and consumers of maternity care. This has resulted in difficulties in evidence uptake, translation and acceptability by those who it is designed to help. Diverse views of ‘what is best’ in research interventions and approaches exist, yet arguably, if consensus can be reached, more rigorous and acceptable interventions will ensue. One such area of uncertainty is hydration management in induced labour, with an increasing reliance on intravenous therapy, without high‐quality evidence pervasive across maternity settings. Therefore, we aimed to co‐design a labour and childbirth intervention to optimise maternal physiology during labour, through evidence synthesis, consumer survey and a consensus‐generating activity.

**Methods:**

A multi‐modal, intervention co‐design study was undertaken. This involved three key stages: evidence‐synthesis, consumer‐survey and then a modified nominal group technique workshop to reach consensus on the intervention design. The whole process took place between April and November, 2023 in South‐East Queensland, Australia.

**Results:**

In total, 96 completed responses were returned in the consumer survey, detailing women's preferences for hydration management during induced labour. This informed a Nominal Group Technique Workshop, inclusive of consumers, midwives, obstetricians, lactation consultants, dietitians, clinical trialists, neonatologists and nurses. Following robust discussion and various viewpoints presented, the expert reference group decided to support an approach whereby women would self‐determine their own intake during induced labour and that the routine administration of intravenous fluids as a ‘side‐line’ (current ‘standard practice’) to the synthetic oxytocin (labour hormone) infusion for induction of labour would not be routinely administered. In order to provide information/education to women on how best to optimise their hydration, a co‐designed information brochure was developed.

**Conclusion:**

What clinicians and researchers may assume is important as interventions and outcomes in a clinical trial may not always align with the opinions and lived experience of consumers. Incorporating co‐design into trial protocol development has the potential to improve the relevance of research to the target population. This in turn may improve trial feasibility through enhanced recruitment, intervention and treatment adherence, and fidelity.

**Patient or Public Contribution:**

People with lived experience (inclusive of both first‐time mothers who had recently experienced a labour induction; and two maternity consumers involved in advocacy and research work to optimise labour and birth care) were involved in several parts of this study. This included study conceptualisation, data collection and analysis, interpretation and authorship of manuscript.

## 1 Background

### 1.1 Maternal Hydration in Labour

Maintaining maternal hydration is an important mechanism to support physiological wellbeing and labour progress [1]. However, it is not well understood, especially for those women undergoing induction of labour, where they are known to experience a longer labour [2] with multiple interventions [3].

During the 1950s, the recognition of Mendelson's syndrome [4] led to many women being routinely fasted during labour and birth, and thus the emergence of intravenous therapy as the key mechanism to maintain hydration [5], and optimise uterine functionality. When women are fasted during spontaneous labour the use of intravenous fluids may reduce labour length [1]. However, fasting during labour is no longer recommended when women have their labours induced [6] and within this context intravenous therapy has not been shown to afford improvements in either labour length or mode of birth [7].

The use of intravenous fluids is now almost ubiquitous across labour and birth settings in high‐resource settings [8, 9], with significant variation in this practice [10] and poor documentation in patient health records [10]. For women undergoing induction of labour, no clear guidelines exist [11] on how best to titrate the intravenous therapy with oral intake to optimise physiology. Evidence is also emerging that intravenous fluids are associated with unintended neonatal harms, such as excessive newborn weight loss [12] or maternal complications, such as breast engorgement or caesarean section [13]. Experimental studies are needed to ascertain how best to maintain maternal euvolaemia during labour to balance physiological needs, maternal comfort and reduce the opportunity for iatrogenic harm.

Women's (consumer) perspectives on this practice are largely unknown. A limited number of studies have been conducted which describe women's oral intake and hydration preferences during labour. McDermott et al. [14] conducted a single‐site study to explore women's preferences and practices for oral intake during labour and birth. They found that most women (*n* = 122; 83%) felt like drinking in labour, despite some nausea and vomiting [14]. Women reported receiving variable advice and would prefer to have clearer recommendations regarding oral hydration during labour. Singata and colleagues' systematic review and meta‐analysis did not identify any harms associated with women eating and drinking during spontaneous labour, and therefore recommend that women are not routinely fasted during labour [15]. However, women undergoing induction of labour or with medical or pregnancy complexities were excluded from this review and thus there is still limited evidence to guide practice for this group.

### 1.2 Why Co‐Design?

In 2022, the Australian Government Department of Health implemented the National Clinical Trials Governance Framework [16] stating healthcare organisations are to develop, implement and maintain systems that ensure healthcare consumers are partners in all aspects of clinical research. Overall, codesigning maternity and perinatal trials is becoming increasingly common, however, how involvement and authentic co‐design is achieved and enabled in busy healthcare settings is less clear. Furthermore, reports on codesigning labour and childbirth interventions are absent in the literature.

Arguably, involving end‐users in research (inclusive of front‐line healthcare providers and maternity consumers) can be challenging. Building consensus from the often‐diverse views and needs of differing disciplines, and those of consumers can be hard. However, when researchers are committed to ensuring all voices are equally privileged, a stronger foundation for research aims and process can be established.

In order to address the uncertainty on how best to maintain maternal hydration during labour, we aimed to co‐design a labour and childbirth intervention to optimise maternal physiology during labour, through evidence synthesis, consumer survey and a consensus‐generating activity. The outcomes will be used to plan a future clinical trial.

## 2 Study One: Consumer Survey

### 2.1 Aim

This study aimed to describe the experiences and hydration practices of pregnant women having their first baby, undergoing induction of labour.

### 2.2 Research Methods

A cross‐sectional survey was undertaken with Australian women who had birthed within the previous 6 months and had their labour induced. Participation was voluntary, and participants were free to withdraw at any time. Participants were invited through social media platforms, such as Facebook, and maternity consumer networks.

Data were collected via an online survey (Qualtrics) between March and May 2023. The survey was designed by the research team, inclusive of a consumer, as no pre‐existing measure was available. The consumer reviewed the survey for face validity. There were 23 questions focused on how women experienced hydration during induced labour. A final ‘free text’ question was included ‘Is there anything else you'd like to tell us about your experience of eating and drinking in labour or having an IV drip during labour?’ The survey assessed participant characteristics, women's preferences and experience of both nutrition and hydration during labour. Women were also asked how they might maintain hydration with their next birth and if the proposed method of hydration the research team were considering would be acceptable (packaged carbohydrate drinks used in the pre‐operating/surgery setting). It was not possible to estimate with any precision the number of women who were eligible to participate in the survey, due to the reach and variation of the potential group.

Data were analysed descriptively using measures of central tendency, as appropriate. Short answer responses to text questions were analysed through content analysis [17]. The study was ethically reviewed and approved by the Metro North Health Human Research Ethics Committee [2023/HE000249].

### 2.3 Results

A total of 122 women responded. In total, 26 were ineligible (labour onset spontaneous *n* = 16; birthed > 6 months ago *n *= 9; birth via elective caesarean section *n *= 1). A total of 96 responses were included in the final analysis. All participants identified as women and were aged 24–44 years old. Women from every state and territory in Australia were represented (QLD = 28; NSW/ACT = 23; VIC = 15; WA = 13; SA = 8; TAS = 4; NT = 3; Missing = 2). All respondents had a support person with them in labour, with this most frequently being their partner (83%; 89) or mother (9%; 10).

Once regular painful contractions had commenced most women continued to drink (74%; 67), with clear fluids preferred, such as water, ice chips, apple juice and coconut water. Of those who chose to eat (72% *n* = 69), food preferences included light sandwiches, fruit, lollies, salty crackers and chips, muesli bars and nuts.

**Figure 1:**
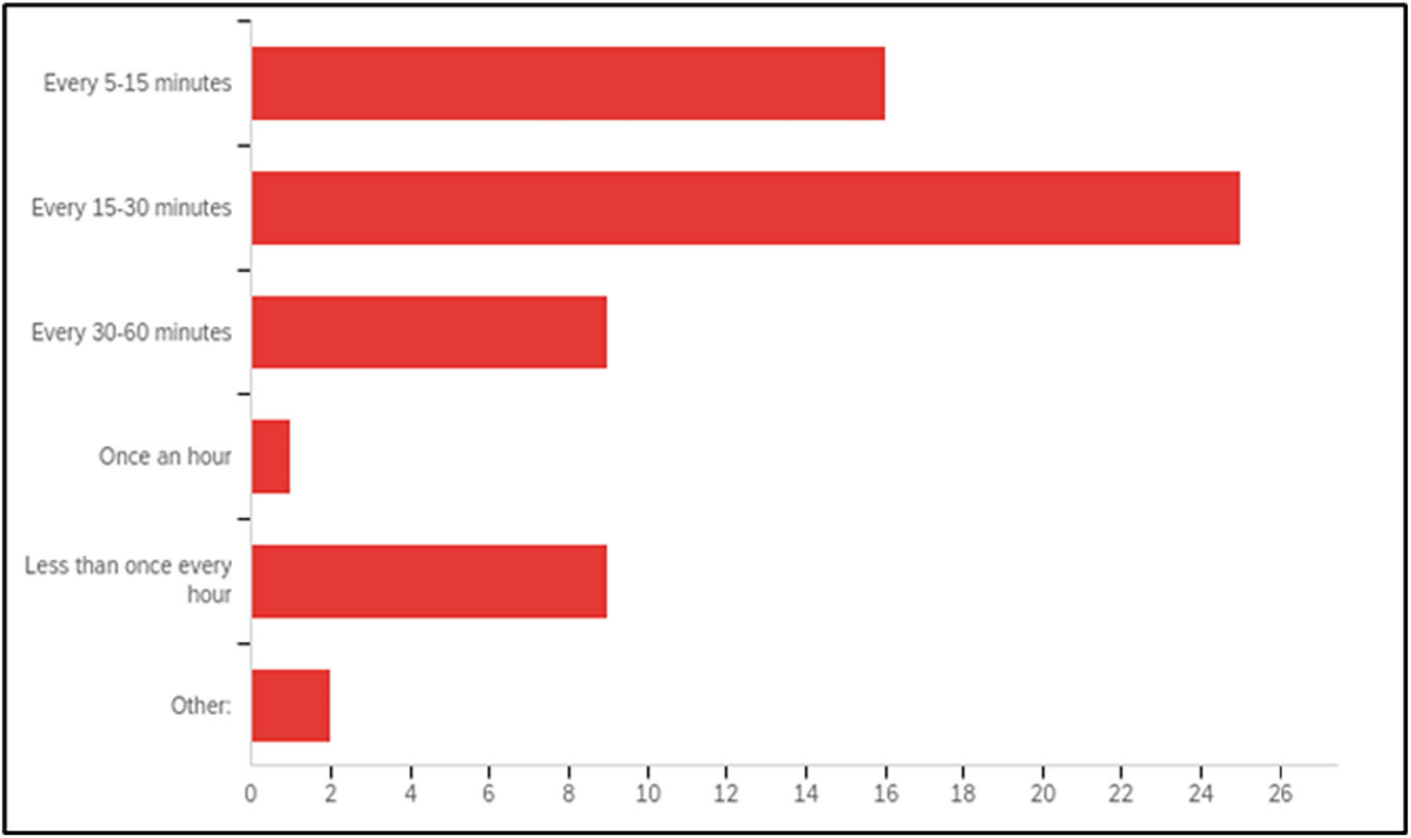
Frequency of drinking in established labour (number).

Drinking patterns were described as small, frequent sips (see Figure 1).

For those who did not drink during established labour, various reasons were given, including not being thirsty (9; 26%), not being offered drinks (6; 17%), and feeling nauseous (7; 20%). Some women described their labour as very rapid, thus limiting time and attention to ingesting food or fluids.

As our participant cohort had experienced induction of labour, we also wanted to understand their experience of labouring with an intravenous infusion/s (‘drip’) running. Four key categories were constructed from women's responses to free text questions about their experiences of labouring with a ‘drip’, and how they perceived the approaches to hydration they had tried. Inductive thematic analysis was the qualitative data analysis approach utilised here [18]. These categories were: Movement was restricted; Opportunity for unintended harm; Safe and reassured; and Partner has a key role (see Table 1).

**Table 1:** Participant feedback on hydration management during induced labour.

Key area	Direct text
Movement was restricted	…unable to move freely or shower for pain relief. I was attached to a IV pole for the induction drugs anyways, so the fluids didn't change much. The IV pole was super annoying and made it more challenging for me to move in and out of the shower (my preferred location to labour). It was another thing attached to me and I had so many. I counted 7 things attached at one point. Minimal effect, though frustrating when pushing having to keep my wrist straight so as not to occlude the line.
Opportunity for unintended harm	The saline and the syntosine [sic] bags accidentally got put through the wrong boxes [IV pumps] so I ended up with 100 mg instead of 10 mg of syntosine [sic] in 1 h, this made the labour quick and intense and made me throw up everything I ate and drank and I ended with and emergency c section. The drip itself didn't really bother me. Annoying and painful as it was placed in my dominant hand. My hand became swollen and tight with fluid and was difficult to move.
Safe and reassured	I think it was good that I had fluids via iv as my labour progressed quickly and intensely from syntocin so the last thing I would want is to eat or drink, however, my body needed to stay hydrated. I'm all for the iv when induced to prevent dehydration. I was very drained, therefore it was essential for me. I struggled to keep food and liquids down.
Partner has a key role	Really helped with hydration, if my partner wasn't encouraging water, I would not have even thought about drinking.

Finally, we were interested in whether women would find ingesting a carbohydrate‐loaded drink (similar to flat lemonade), such as those used within the Enhanced Recovery After Surgery literature [19] acceptable. Women in our survey favoured a self‐determined approach in choosing which fluids they would ingest, with carbohydrate drinks (mean 63.90 (Scale 0–100)) preferred less often to ‘other’ self‐determined drinks (mean 79.50 (Scale 0–100)).

## 3 Study Two: Consensus Activity

The second study aimed to develop a maternal hydration intervention, co‐designed by consumers and practising clinicians.

### 3.1 Research Methods

A Nominal Group Technique (NGT) [20] activity was undertaken to establish the oral hydration intervention. During November 2023 the NGT was held with an expert reference group conducted over 3 h. Participation was purposefully sampled to ensure adequate representation including: Obstetricians (*n* = 2); Senior midwives (*n* = 4); Dietician (maternal health) (*n *= 1); Clinical trialists (*n *= 2); Consumers (*n* = 2); Neonatologist (*n* = 1); Neonatal nurse (*n* = 1); International board‐certified lactation consultant (*n* = 1), Total (*n* = 14). (The Anaesthetist involved in the group was unable to attend on the day but reviewed the co‐designed outcome intervention and agreed with the broader group).

The NGT applies a consensus‐based method, involving four main stages:

1. Silent generation: a brief presentation was provided by L. K. (who has been involved in leading two NGT before) to the group summarising the consumer survey results and feedback, and the results from the evidence synthesis. The group was then provided time to quietly consider this before step 2. Questions were posed, such as ‘Would you find the carbohydrate drink palatable in labour?’ (including samples of the drink to taste; and ‘what is your experience of maternal hydration behaviours and tolerability in labour’?)
2. Round robin: each participant was invited to share their experience and perspective regarding managing maternal hydration in labour, either from their own lived experience or that of a clinician providing care and guidance.
3. Clarification: during this stage a draft protocol for maintaining oral hydration was drafted in consultation with the group. Any areas of ambiguity were discussed and clarified.
4. Voting (ranking): See below, but conducted post‐workshop [21].

**Figure 2:**
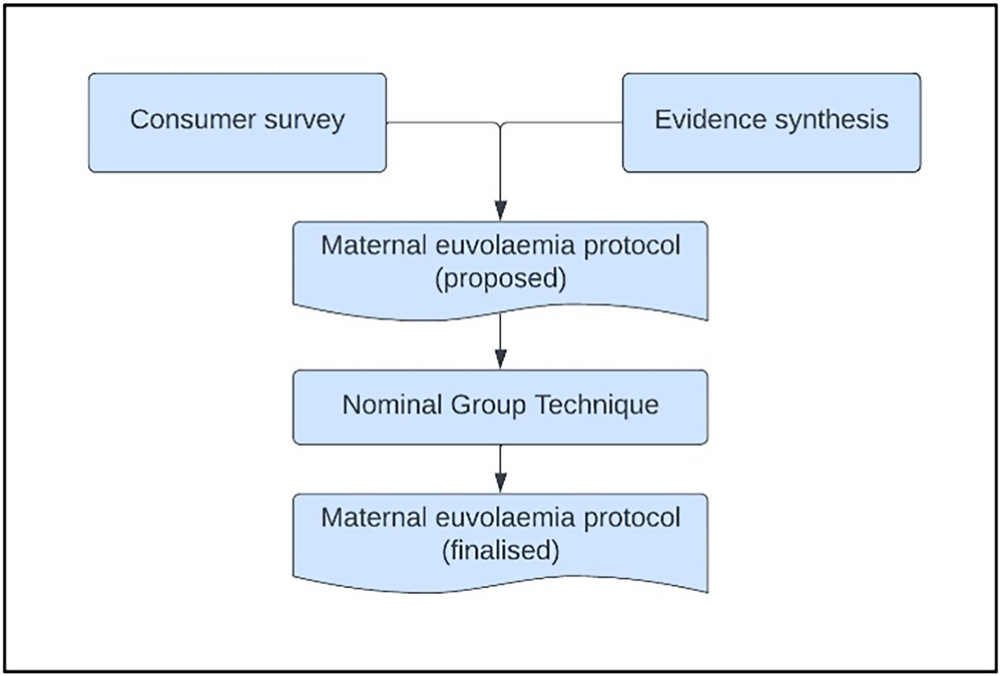
Intervention development process.

Originally developed by Van de Ven and Delbecq [22], this approach facilitated time for individual reflection and contributions, with ‘turn taking’ emphasised, thus being a good choice to privilege voices of those who may be less confident ‘speaking up’‐ such as consumers. As seen in Figure 2, both the knowledge and perspectives of participants and the evidence synthesis already conducted (scoping review of clinical guidelines [11] and systematic review of intravenous hydration for women undergoing induction of labour [7]) were considered by the group to develop an intervention protocol which was then discussed and agreed upon (this can be found in detail by accessing the Australia and New Zealand Clinical Trials Registry (ACTRN12623001137684). This is a slight modification from traditional NGT process.

The process outlined in Figure 2 was adopted. Following the presentation of synthesised literature and ‘round robin’ stages, all participants were afforded opportunity to ask questions and discuss differences (clarification stage). The investigators then summarised the key topics and presented a proposed protocol for ranking by the group. This occurred via email, following the workshop, as the clinical demands of some group members restricted the time they had to participate. Consensus was set, a priori, at 80% agreement [20], however, the group obtained a 100% agreement.

### 3.2 Results

Following robust discussion and various viewpoints presented, the expert reference group decided to support an approach whereby women would self‐determine their own intake during induced labour and that the routine administration of intravenous fluids as a ‘side‐line’ to the syntocinon (synthetic oxytocin hormone medicine that causes uterine contractions) infusion for induction of labour would not be routinely administered. In order to provide information/education to women on how best to optimise their hydration, a co‐designed information brochure was developed. The brochure drew from the recommendations women provided in the survey as to what they preferred to eat and drink in labour as its fundamental starting point. This content was then reviewed by our lead maternity dietitian in terms of suitability, our lead anaesthetist, midwifery and obstetric members and edits made as required. Information was phrased consistently with consumer concerns relating to hydration in labour too, including sections on ‘Is it safe to eat and drink during labour?’ and ‘what happens if I feel sick or vomit’? Evidence and practical solutions were also incorporated, for example, suggestion to drink small amounts, often, to reduce likelihood of vomiting and ensuring a range of clear fluids were included (as some women may be recommended to only ingest these if a caesarean section is looking likely to occur during labour). This brochure, alongside a brief midwifery education session, has now been piloted in our feasibility clinical trial [for details, see ANZCTR Trial ID: ACTRN12623001137684]. These results will be published elsewhere.

## 4 Discussion

Ensuring intervention development occurs within a context of consumer and end‐user (clinician) engagement is an important component of high‐quality applied research. This paper has demonstrated one pragmatic exemplar of how this process can be undertaken in a collaborative and collegial way and is intended to share this process with others. Our initial hypothesis to use pre‐operative drinks as an adjunct to maintain hydration in labour was not supported by consumers and therefore the research team adapted the intervention, accordingly, instead providing guidance to women, and supporting them to maintain self‐determination over their oral hydration in labour. Previous reviews have found that whilst research co‐design is reported to be widely used, it is seldom described or evaluated in detail [23]. Therefore, our study aims to address this deficit, through the reporting of the activities taken to design the intervention of our future trial.

### 4.1 Involving Consumers in Trial Design

Recruiting pregnant women to interventional studies, especially when the intervention occurs during the labour and childbirth period, can be challenging. Historically, women have been underrepresented in clinical trials, especially those involving new treatments or devices. Concerns have been raised regarding the lack of involvement of women as participants in clinical trials [24], which has led to a lack of high‐quality evidence to guide much of the clinical care we provide to women, especially during the childbearing period.

Furthermore, difficulties and challenges have been reported in the recruitability and subsequent randomisation of women for research during pregnancy and labour and childbirth care [25]. Non‐compliance with the protocol [25], challenges with consent during established labour [26] with a reliance on clinical staff to undertake recruitment, and the changing ‘risk’ profile can create challenges for high‐quality research to be undertaken. In response, our work offers a potential solution to these challenges through consumer involvement and engagement from the very beginning of trial design. Further considerations and research in how to further optimise engagement from even more marginalised groups (such as culturally and linguistically diverse and socioeconomically disadvantaged peoples) are needed.

### 4.2 Strengths and Limitations

This study highlights a pragmatic approach to intervention design for a planned clinical trial involving pregnant women having their first baby, undergoing induction of labour. With minimal existing literature, the evidence‐synthesis component was limited, and more research is urgently needed. We acknowledge therefore that our protocol is largely based on consensus, and that consensus‐based approaches can be limited by the subjective views of the participants and lead to potential bias. Our initial consultation survey was relatively small (*n* = 96) and the views expressed may not be representative of all potential participants in future experimental studies applying the intervention. In total, 21% of women who completed the survey were ineligible as their labour was not induced, and whilst their views are important, clear guidelines already exist on oral intake for their circumstance [11].

## 5 Conclusion

What clinicians and researchers may assume is important as interventions and outcomes in a clinical trial may not always align with the opinions and lived experience of consumers. Incorporating co‐design into trial protocol development has the potential to improve the relevance of research to the target population. This in turn may improve trial feasibility through enhanced recruitment, intervention and treatment adherence, and fidelity.

## Author Contributions

**Lauren Kearney:** conceptualisation, methodology, formal analysis, writing – review and editing. **Bec Jenkinson:** conceptualisation, methodology, writing – review and editing. **Nigel Lee:** formal analysis, writing – review and editing. **Christoph Lehner:** writing – review and editing. **Victoria Eley:** writing – review and editing. **Nicole Marsh:** writing – review and editing. **Deanne August:** writing – review and editing. **Susan de Jersey:** writing – review and editing.

## Ethics Statement

This study had ethical approval from both the public health service [HREC/2023/MNHA/97081] and the university [2023/HE001417].

## Consent

All individuals who participated in this study provided informed consent.

## Conflicts of Interest

D.A. has undertaken consultancy work for NAVI technologies unrelated to this study. The other authors declare no conflicts of interest.

## Data Availability

Data collected for this study only sought ethics approval for use within this specific study, therefore, original data cannot be shared with other researchers.

## References

[1] F. Dawood , T. Dowswell , and S. Quenby , “Intravenous Fluids for Reducing the Duration of Labour in Low Risk Nulliparous Women,” Cochrane Database of Systematic Reviews 2013, no. 6 (2013): CD007715, 10.1002/14651858.CD007715.pub2. 23780639 PMC11650501

[2] D. Coates , A. Makris , C. Catling , et al., “A Systematic Scoping Review of Clinical Indications for Induction of Labour,” PLoS One 15, no. 1 (2020): e0228196. 31995603 10.1371/journal.pone.0228196 PMC6988952

[3] H. G. Dahlen , C. Thornton , S. Downe , et al., “Intrapartum Interventions and Outcomes for Women and Children Following Induction of Labour at Term in Uncomplicated Pregnancies: A 16‐Year Population‐Based Linked Data Study,” BMJ Open 11, no. 6 (2021): e047040. 10.1136/bmjopen-2020-047040 PMC8169493 34059509

[4] C. L. Mendelson , “The Aspiration of Stomach Contents Into the Lungs During Obstetric Anesthesia,” American Journal of Obstetrics and Gynecology 52 (August 1946): 191–205. 20993766 10.1016/s0002-9378(16)39829-5

[5] The Amercian College of Nurse‐Midwives , Providing Oral Nutrition to Women in Labor: American College of Nurse‐Midwives,” Journal of Midwifery & Women's Health 61, no. 4 (July 2016): 528–534. 10.1111/jmwh.12515 27383919

[6] D. J. Lowen , N. Carlon , and R. Hodgson , “A Survey of Australian Midwifery Intravenous Fluid Management During Induction of Labour,” Collegian 30 (August 2023): 640–646.

[7] L. Kearney , S. Brady , N. Marsh , M. Davies‐Tuck , R. Nugent , and V. Eley , “The Effects of Intravenous Hydration Regimens in Nulliparous Women Undergoing Induction of Labor: A Systematic Review and Meta‐Analysis,” Acta Obstetricia et Gynecologica Scandinavica 103 (March 2024): 1254–1262. 38468190 10.1111/aogs.14793 PMC11168270

[8] L. Kearney , A. Craswell , D. Massey , et al., “Peripheral Intravenous Catheter Management in Childbirth (PICMIC): A Multi‐Centre, Prospective Cohort Study,” Journal of Advanced Nursing 77, no. 11 (November 2021): 4451–4458. 34118163 10.1111/jan.14933

[9] D. J. Lowen , P. Howat , and R. Hodgson , “An Australian and Aotearoa New Zealand Audit of Obstetric Fluid Management During Induction of Labor,” Journal of Clinical Gynecology and Obstetrics 12, no. 3 (December 2023): 71–77.

[10] B. R. Bruce , D. L. Hartz , S. K. Tracy , J. Leask , and B. S. de Vries , “The Administration of Intravenous Fluids to Nulliparous Women in Labour: A Retrospective Clinical Chart Review and Fluid Balance Documentation Audit,” Collegian 29, no. 3 (2022): 364–369.

[11] L. Kearney , A. Craswell , N. Dick , D. Massey , and R. Nugent , “Evidence‐Based Guidelines for Intrapartum Maternal Hydration Assessment and Management: A Scoping Review,” Birth 51, no. 2 (June 2023): 253–263, 10.1111/birt.12773. 37803945

[12] M. Rich , S. Dowling , and I. Bray , “Maternal Intrapartum Fluids and Neonatal Weight Loss in the Breastfed Infant,” British Journal of Midwifery 31, no. 6 (2023): 344–351.

[13] S. Kujawa‐Myles , J. Noel‐Weiss , S. Dunn , W. E. Peterson , and K. J. Cotterman , “Maternal Intravenous Fluids and Postpartum Breast Changes: A Pilot Observational Study,” International Breastfeeding Journal 10, no. 1 (2015): 18. 26113871 10.1186/s13006-015-0043-8 PMC4480510

[14] L. McDermott , A. Pelecanos , A. Krepska , et al., “Single‐Centre Survey of Women Reflecting on Recent Experiences and Preferences of Oral Intake During Labour,” Australian and New Zealand Journal of Obstetrics and Gynaecology 62, no. 5 (2022): 643–649. 35342926 10.1111/ajo.13509

[15] M. Singata , J. Tranmer , and G. M. Gyte , “Restricting Oral Fluid and Food Intake During Labour,” Cochrane Database of Systematic Reviews 2013, no. 8 (2013): CD003930, 10.1002/14651858.CD003930.pub3. 23966209 PMC7104541

[16] Australian Commission on Safety and Quality in Health Care , The National Clinical Trials Governance Framework and User Guide for Health Service Organisations Conducting Clinical Trials (ACSQHC, 2022).

[17] S. Elo and H. Kyngäs , “The Qualitative Content Analysis Process,” Journal of Advanced Nursing 62, no. 1 (2008): 107–115. 18352969 10.1111/j.1365-2648.2007.04569.x

[18] V. Braun and V. Clarke , “Using Thematic Analysis in Psychology,” Qualitative Research in Psychology 3, no. 2 (2006): 77–101.

[19] M. Melnyk , R. G. Casey , P. Black , and A. J. Koupparis , “Enhanced Recovery After Surgery (ERAS) Protocols: Time to Change Practice?,” Canadian Urological Association Journal 5, no. 5 (2011): 342–348. 22031616 10.5489/cuaj.11002 PMC3202008

[20] N. Harvey and C. A. Holmes , “Nominal Group Technique: An Effective Method for Obtaining Group Consensus,” International Journal of Nursing Practice 18, no. 2 (April 2012): 188–194. 22435983 10.1111/j.1440-172X.2012.02017.x

[21] S. S. McMillan , M. King , and M. P. Tully , “How to Use the Nominal Group and Delphi Techniques,” International Journal of Clinical Pharmacy 38, no. 3 (2016): 655–662. 26846316 10.1007/s11096-016-0257-x PMC4909789

[22] A. H. Van de Ven and A. L. Delbecq , “The Nominal Group as a Research Instrument for Exploratory Health Studies,” American Journal of Public Health 62, no. 3 (March 1972): 337–342. 5011164 10.2105/ajph.62.3.337 PMC1530096

[23] P. Slattery , A. K. Saeri , and P. Bragge , “Research Co‐Design in Health: A Rapid Overview of Reviews,” Health Research Policy and Systems 18, no. 1 (2020): 17. 32046728 10.1186/s12961-020-0528-9 PMC7014755

[24] Women's Health Victoria, *Women in Clinical Trials*, https://www.whv.org.au/resources/whv-publications/women-clinical-trials#:~:text=Women%20have%20been%20historically%20underrepresented,often%20not%20analysed%20and%20reported.

[25] V. Hundley and H. Cheyne , “The Trials and Tribulations of Intrapartum Studies,” Midwifery 20, no. 1 (March 2004): 27–36. 15020025 10.1016/S0266-6138(03)00050-0

[26] S. L. Hansen , S. L. Clark , and J. C. Foster , “Active Pushing Versus Passive Fetal Descent in the Second Stage of Labor: A Randomized Controlled Trial,” Obstetrics and Gynecology 99, no. 1 (January 2002): 29–34. 11777506 10.1016/s0029-7844(01)01642-8

